# Protective outcomes of high-affinity monoclonal antibodies against drug-resistant plague strains

**DOI:** 10.3389/fimmu.2026.1835695

**Published:** 2026-06-23

**Authors:** Wenxuan Peng, Hailian Wu, Li Zhang, Binyang Zheng, Qi Zhang, Wenyuan Xin, Xiaoyan Yang, Kaiye Ying, Ru Ma, Hongxing Pan, Qingwen Zhang, Haisheng Wu

**Affiliations:** 1 Qinghai Institute for Endemic Diseases Prevention and Control, Key Laboratory for Plague Prevention and Control of Qinghai Province, Xining, Qinghai, China; 2 NHC Key Laboratory of Plague Prevention and Control, Qinghai Institute for Endemic Disease Prevention and Control, Xining, Qinghai, China; 3 Jiangsu Provincial Center for Disease Prevention and Control, Department of Vaccine Clinical Evaluation, Nanjing, Jiangsu, China; 4 National Vaccine Innovation Platform, Nanjing Medical University, Nanjing, China

**Keywords:** drug-resistant infection, efficacy assessment in animal models, novel anti-plague monoclonal antibodies, novel treatment modalities for plague, *Yersinia pestis*

## Abstract

**Background:**

*Yersinia pestis*, a category A infectious organism, is known to cause bubonic, septicemic, and pneumonic plague. With the emergence of streptomycin-resistant strains, there is an urgent need for new therapeutic strategies that can protect populations from *Y. pestis* infection. The main strategy for developing vaccines and therapeutic antibodies involves F1 antigen. Previous research has demonstrated that both human and murine monoclonal antibodies(mAbs) confer protective effects against *Y. pestis* infection. While, no relevant studies were identified on mAbs against drug-resistance *Y. pestis*.

**Methods:**

Here, we constructed an antibody library from *Y. pestis* vaccine strain EV76-immunized mice by phage display. The mAbs were baited by recombinant F1 antigen, and the biological functions of the obtained mAbs were assessed and evaluated in plague-challenged mice.

**Results:**

For the *Y. pestis* strain 141 group, both Fm3 and Fm25 provided 100% protection at a dose of 100 µg. At 20 µg, only Fm3 conferred partial protection, with a survival rate of 25%, whereas all mice in the 4 µg treatment groups succumbed to infection within approximately 10 days. Against the drug-resistant *Y. pestis* strain S19960127, Fm25 at 100 µg resulted in complete survival, whereas Fm3 at the same dose conferred 75% protection. Neither mAbs showed protective efficacy at 20 µg or 4 µg, and all animals dying within approximately 10 days post-infection.

**Conclusion:**

These findings indicated that mAbs Fm3 and Fm25 confer protection against virulent and drug-resistant strains of *Y. pestis*.

## 1 Introduction

*Y. pestis* is the causative agent of plague, an infectious disease of historical and contemporary significance that is infamous for three devastating pandemics resulting in millions of fatalities (1–3).Although the modern public health system has effectively curbed large-scale plague epidemics, many countries and regions in Africa, Asia, and the Americas have become major natural foci and active epidemic areas (4–6). This situation poses a persistent challenge to international public health efforts and global biosafety governance (7, 8).Traditional approaches to plague management encompass multifaceted strategies involving rodent control, flea eradication, vaccination, and antibiotic treatment (9–12). Earlier vaccination strategies utilized inactivated and live attenuated vaccines; however, contemporary approaches primarily focus on recombinant subunit vaccines incorporating the F1 and V antigens, which are now regarded as the standard of care (13–16). The F1 antigen, a key virulence factor of *Y. pestis*, forms a capsule that inhibits phagocytosis, suppresses TLR/NF-κB signaling, and facilitates biofilm formation. Anti-F1 antibodies provide protective effects through multiple mechanisms. For example, it could enhance macrophage-mediated opsonophagocytosis, block the type III secretion system (T3SS) to prevent translocation of *Yersinia* outer protein (Yop) effectors, and activate complement cascades and neutrophils via the Fragment crystallizable(Fc) region (17–20). Despite the general susceptibility of *Y. pestis* to antibiotics, antimicrobial therapy remains the cornerstone of plague management (21, 22), as standardized in several authoritative guidelines, including the World Health Organization (WHO) plague manual (23), the U.S. Centers for Disease Control and Prevention (CDC) treatment recommendations (24), the European Medicines Agency (EMA) guidelines (25), and China’s “Plague Diagnosis and Treatment Scheme (Trial) (26).” Streptomycin, tetracyclines, and chloramphenicol continue to serve as the primary agents within these recommended regimens. However, the emergence of a multidrug-resistant *Y. pestis* strain in Madagascar has underscored the significant limitations of antibiotic therapy (27–29).Furthermore, it poses a significant challenge to the existing infectious disease prevention and control systems (30, 31). Monoclonal antibodies (mAbs), with their dual utility in both prevention and treatment, have demonstrated substantial therapeutic benefits across multiple disease models (32–34). Currently, no approved mAbs therapies exist for the treatment of plague. Previously, our research group reported that three human mAbs could protect BALB/c mice against lethal challenge with the virulent *Y. pestis* 141 (35).However, the protective efficacy of mAbs against drug-resistant bacterial strains had not been evaluated in that prior study. In the present investigation, we derived two mAbs designated Fm3 and Fm25 from mice immunized with *Y. pestis* EV76. These two mAbs could protect BALB/c mice against infection with both the virulent *Y. pestis* 141 and a streptomycin-resistant strain. Consequently, Fm3 and Fm25 provide valuable candidates and alternative therapeutic approaches for treating drug-resistant *Y. pestis* infection.

## 2 Materials and methods

### 2.1 Bacterial strains and antigens

The three *Y. pestis* strains used in this study were isolated and maintained at the Qinghai Institute for Endemic Disease Prevention and Control. The vaccine strain EV76 is a live attenuated *Y. pestis* derivative and is also commonly used to evaluate the protective efficacy in plague mouse models (36). The *Y. pestis* strain 141, originally isolated from Marmota himalayana in the Qinghai-Tibet Plateau, exhibits strong virulence in both humans and mice, with a median lethal dose (MLD) of 5.6 colony-forming units (CFU) for BALB/c mice (37, 38). The drug-resistant *Y. pestis* strain S19960127 was isolated from the autopsy organs of a plague patient. In 2021, researchers found that the strain was highly resistant to streptomycin, with a minimum inhibitory concentration (MIC) of 4,096 mg/L (39). The recombinant F1 antigen of *Y. pestis* was supplied by the Lanzhou Institute of Biological Products Co., Ltd.

### 2.2 Animal experiments

Female BALB/c mice of 6–8 weeks were obtained from Xi’an Huishi Biotechnology Co., Ltd. [SCXK(YU)2020-0005]. The animal study protocol was reviewed and approved by the Institutional Animal Care and Use Committee (IACUC) of the Qinghai Institute for Endemic Disease Prevention and Control (QDB21-2504-002). All experimental procedures were performed in accordance with the institution’s relevant guidelines and regulations.

### 2.3 Reagents and plasmids

First-strand complementary DNA (cDNA) was synthesized using the SuperScript First-Strand Synthesis Kit (Invitrogen). The polymerase chain reaction (PCR) kit was obtained from Takara (Takara Biotechnology, Dalian, China). *Sfi*I restriction enzyme and T4 ligase were purchased from New England Biolabs (NEB). The HEK293F cell line was sourced from the American Type Culture Collection (ATCC). The *Escherichia coli* strains DH5α and XL1-Blue, the phagemid vector pComb3XSS, and antibody expression vectors pGI-mH and pGI-mK were maintained at the Jiangsu Provincial Center for Disease Control and Prevention. All other chemicals used were of analytical grade.

### 2.4 Animal immunization

All experiments were conducted in the Animal Biological Safety Level 2 (ABSL-2) laboratory at the Plague Specialized Laboratory of the Qinghai Institute for Endemic Disease Prevention and Control. A total of six mice were immunized subcutaneously in the inguinal region with 0.5 mL containing 5 × 10^7^ colony-forming units (CFU) of the EV76 strain on days 0, 10, and 20. On day 30 post-immunization, mice were bled for serum collection and subsequently euthanized for spleen removal. F1-specific antibodies in the serum were detected by indirect enzyme-linked immunosorbent assay (ELISA). The spleens were homogenized in a biological safety cabinet to prepare single-cell suspensions. Mononuclear cells were subsequently isolated from splenocytes using Ficoll density gradient centrifugation.

### 2.5 Generating a murine scFv antibody library

Total RNA was extracted from murine peripheral blood mononuclear cells (PBMCs) using the QIAGEN RNeasy Mini Kit, followed by reverse transcription into cDNA using the First-Strand cDNA Synthesis Kit. Primers for the mouse variable region of the antibody heavy chain (VH) and variable region of the antibody kappa light chain (VK) were designed according to the International ImMunoGeneTics information system (IMGT) database (**Supplementary Table 1**).

The VH chains were amplified using a mixture of MVH1 to MVHS2 as the forward primer and a mixture of MSCG1ab-B and MSCG3-B as the reverse primers. Similarly, the VK chains were amplified using MVK1 to MVK12 as the forward primer and a mixture of MSCJK12-B, MSCJK4-B, and MSCJK5-B as the reverse primers. The PCR product was verified by agarose gel electrophoresis, and the target band was recovered using a gel recovery kit. Using overlapping PCR, VH and VK were fused to generate scFv fragments. The purified scFv fragments were digested with SfiI and ligated into the pComb3XSS vector. The ligation mixture was electroporated into XL1-Blue competent cells to generate the primary phage antibody library. Finally, the library was rescued and amplified using wild-type helper phage VCSM13 for subsequent panning and selection.

### 2.6 Panning F1-specific phage antibodies

The antibody library was subjected to panning using *Y. pestis* F1 antigen prepared according to the previously reported method (40). A total of three rounds of biopanning were performed to enrich for F1-specific binders. The phage eluted from the third round of panning was used to infect logarithmic-phase XL1-Blue host bacterial cells. The infected culture was serially diluted and plated on Luria-Bertani (LB) agar plates containing ampicillin. The following day, 96 individual colonies were randomly selected and inoculated into a 96-deep-well plate containing growth medium. The plate was incubated at 37 °C with shaking at 260 revolutions per minute (rpm) for 4 hours. Subsequently, the cultures were induced by adding 1 millimolar (mM) isopropyl β-D-1-thiogalactopyranoside (IPTG), followed by overnight incubation at 37 °C. The next day, the expression of specific antibodies in the culture supernatants was analyzed by indirect ELISA. Briefly, a 96-well microtiter plate was coated with *Y. pestis* F1 antigen at 200 nanograms (ng) per well. Culture supernatant from the deep-well plate was then added to each well. The plate was incubated at 37 °C with shaking for 1 hour. Following three washes with phosphate-buffered saline containing Tween 20 (PBST), a horseradish peroxidase (HRP)-conjugated anti-M13 mAbs was added, and the plate was incubated for 30 minutes at 37 °C. After another wash with PBST, the reaction was developed with tetramethylbenzidine (TMB) substrate for 10 minutes and stopped by adding 2 molar (M) sulfuric acid. The absorbance was measured at 450 nanometers (nm). Plasmids from positive clones identified by ELISA were extracted and submitted for DNA sequencing. The obtained variable region sequences were analyzed and compared using the IMGT database.

### 2.7 Expression of murine IgG in mammalian cells

The VH chain genes were cloned into the pGI-mH vector, which contains the mouse immunoglobulin G2a (IgG2a) constant region, using the *Age*I and *Sal*I restriction sites. Similarly, the VK chain genes were cloned into the pGI-mK vector, which contains the mouse kappa light chain constant region, using the *Age*I and *Bsi*WI restriction sites. The two plasmids were co-transfected into HEK293F cells using polyethylenimine as the transfection reagent. The culture supernatant was harvested five days post-transfection. The target antibody was subsequently purified from the supernatant using a Protein A affinity chromatography column.

### 2.8 ELISA and western blot analysis

The sensitivity and specificity of the mAbs were evaluated by ELISA and Western blot analysis. For the indirect ELISA, a 96-well microtiter plate was coated with F1 protein at 1 microgram per milliliter (µg/mL). The purified antibodies, starting at a concentration of 100 ng/mL, were subjected to two-fold serial dilutions. The diluted antibodies were added to the plate and incubated at 37 °C for 1 hour. After washing with PBST, an HRP-conjugated goat anti-mouse immunoglobulin G (IgG) secondary antibody was added, followed by a 30-minute incubation at 37 °C. The color reaction was developed with TMB substrate and stopped, after which the absorbance at 450 nm was measured. All samples were analyzed in triplicate, and mean values were calculated. Subsequently, Western blot analysis was performed to confirm binding specificity. Briefly, 10 µg of F1 protein was separated by sodium dodecyl sulfate polyacrylamide gel electrophoresis (SDS-PAGE) and electroblotted onto a polyvinylidene fluoride (PVDF) membrane. The membrane was blocked with 3% nonfat milk and then incubated with the mAbs. Following washes, the membrane was incubated with an HRP-conjugated goat anti-mouse IgG secondary antibody. Finally, enhanced chemiluminescence (ECL) was used to develop and visualize the image.

### 2.9 Binding affinity analysis

The affinity constant and binding kinetics of the murine mAbs against the F1 antigen were determined using bio-layer interferometry (BLI) on a Sartorius Octet R8 molecular interaction analysis system. Briefly, the antibody was immobilized onto protein A (ProA) biosensors. Following a baseline stabilization step, the antibody-loaded sensors were exposed to a series of serially diluted F1 antigen solutions. Binding interactions were monitored in real-time by measuring shifts in the interference pattern of reflected light. Data regarding molecular interactions and binding kinetics, including the association rate constant (ka), dissociation rate constant (kd), and equilibrium dissociation constant (KD), were collected and analyzed throughout the measurement process.

### 2.10 Antibody binding competition assay

An antibody binding competition assay was performed using BLI on Octet Red96 instrument. Biotinylated F1 was loaded onto streptavidin (SA) sensors (ForteBio Cat# 18-5019) to a load threshold of 1.5 nm. After a baseline step, the first mAbs (Fm3 or Fm25) at 15 µg/ml were associated to sensor for 180 seconds. Only Fm25 was used as the secondary antibody at 15 µg/ml. The biosensors were immersed into Fm25 to record binding competition data with first mAbs.

### 2.11 Preparation of highly virulent *Y. pestis* 141 and S19960127 bacterial suspensions

The standard virulent *Y. pestis* 141 and S19960127 were resuscitated and cultured. Bacterial lawns were harvested and homogenized in normal saline to prepare a primary suspension. The turbidity of this suspension was adjusted to a concentration of 1 × 10^8^ colony-forming units per milliliter (CFU/mL). The primary suspension was subsequently serially diluted to obtain the following concentrations: 1 × 10^7^, 1 × 10^6^, 1 × 10^5^, 1.5 × 10^4^ (representing 100 MLD), 1 × 10^4^, 1 × 10^4^, 300 (2 MLD), 150 (1 MLD), and 75 CFU/mL (0.5 MLD). For the experimental group, mice were challenged via subcutaneous injection in the inguinal region with 0.5 mL of bacterial suspension at a concentration of 1.5 × 10^4^ CFU/mL. Control groups were challenged using the same route and volume with suspensions containing 1.5 × 10^4^, 300, 150, and 75 CFU/mL. To verify the viable bacterial count in the inoculum, 100 μL aliquots of the 1 × 10^4^ and 1 × 10^4^ CFU/mL suspensions were spread evenly onto Hottinger agar plates. The plates were incubated at 28 °C for 48 hours, after which colonies were enumerated. The actual challenge dose was calculated based on the mean colony count.

### 2.12 Evaluation of the protective efficacy of mAbs against challenge with *Y. pestis* 141 and S19960127 in a mouse model

Mice were divided into groups (n=4) and administered the respective mAbs via intraperitoneal injection 24 hours prior to bacterial challenge. Each antibody was evaluated at three different doses: 100 µg, 20 µg, and 4 µg per mouse. All groups consisted of 6- to 8-week-old female BALB/c mice. One day after antibody administration, all mice were challenged subcutaneously in the inguinal region with 100 MLD of the virulent *Y. pestis* 141. To verify the virulence of the bacterial stock, control groups of mice that did not receive antibody treatment were challenged with 100 MLD, 2 MLD, 1 MLD, and 0.5 MLD of the same virulent strain. The challenge experiment for the *Y. pestis* S19960127 was performed using an identical protocol for grouping, antibody administration, and challenge procedures as described for virulent *Y. pestis* 141 or S19960127 (**Table 1**). Following challenge, all mice were housed in sealed isolator cages and monitored daily for body weight changes and survival. Mice that succumbed to infection were necropsied promptly. Heart, liver, and lung tissues were collected for imprint smearing and cultured on selective media for *Y. pestis* isolation. Culture-positive isolates were subsequently confirmed as *Y. pestis* by a species-specific bacteriophage lysis assay.

**Table 1:** Experimental grouping of passive immunization of mice and attack by *Y. pestis* 141 or S19960127.

mAb	Group	Passive immunotherapy			141 or S19960127(after immunization24h)
		Concentration (μg/mouse)	Vaccination dose(mL)	Route of inoculation	Bacterial concentration(MLD)
Fm3	1	100	0.5	Intraperitoneal	100
	2	20	0.5	Intraperitoneal	100
	3	4	0.5	Intraperitoneal	100
Fm25	1	100	0.5	Intraperitoneal	100
	2	20	0.5	Intraperitoneal	100
	3	4	0.5	Intraperitoneal	100
Naive Control	1	–	–	–	100
	2	–	–	–	2
	3	–	–	–	1
	4	–	–	–	0.5

For all groups, n=4 mice per group. The challenge dose (0.5 mL) was administered by subcutaneous injection in the groin.

### 2.13 Statistical analysis

The observation period was 21.00 days. Mice that survived at the end of the observation period were counted as 21.00 days. Survival rates and mean survival times were calculated from the mortality data. Survival data were analyzed using the Kaplan–Meier method, and the mAbs dose was correlated with mouse survival time using Spearman’s rank correlation.

## 3 Results

### 3.1 High-titer anti-F1 antibody response following EV76 immunization

**Figure 1:**
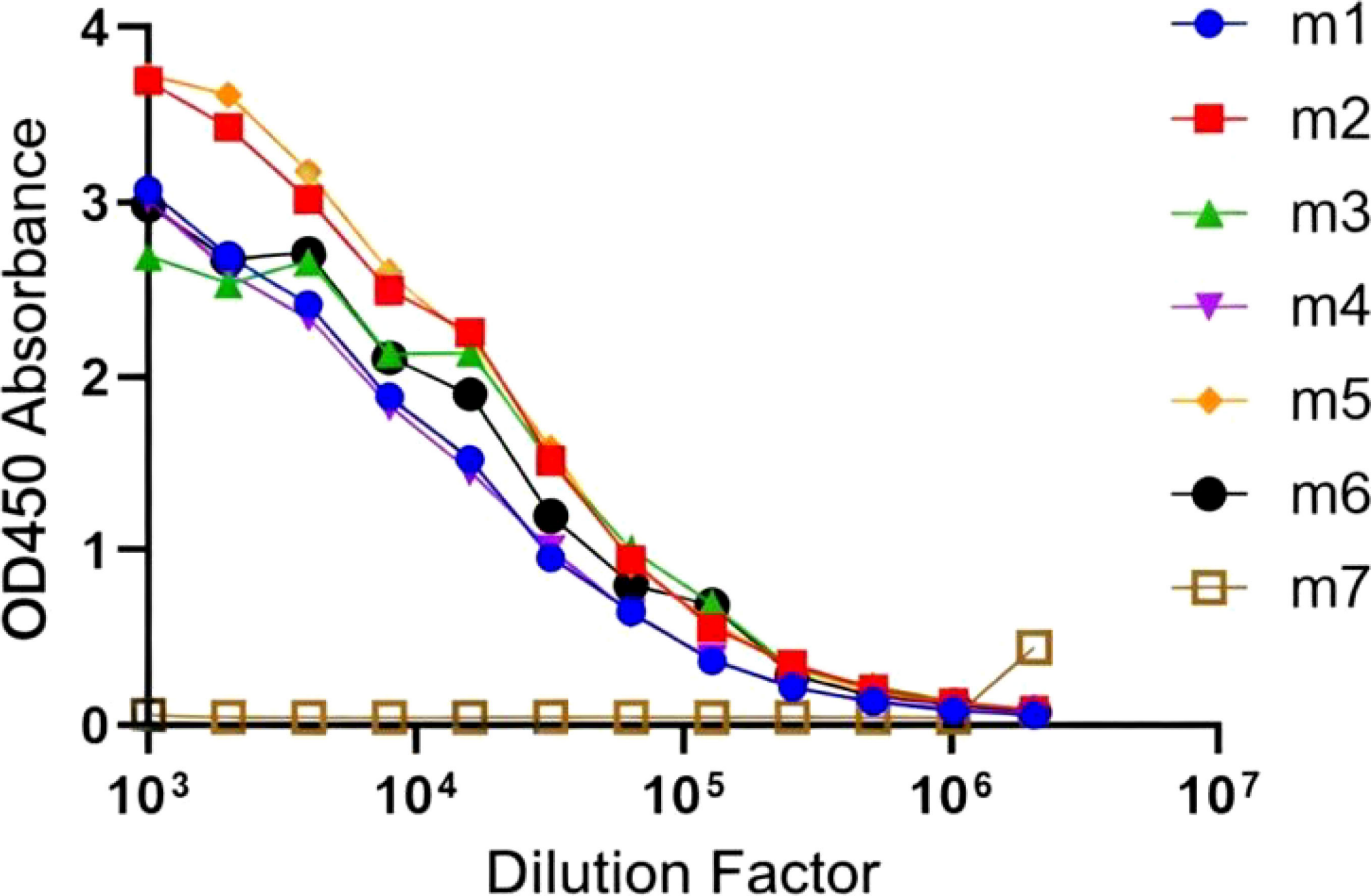
Mice responses against vaccine strain EV76 of *Y. pestis*. Total 5×10^7^ CFU of EV76 strain on days 0, 10, and 20. Animal serum were collected on day 30. Purified F1 antigen coated on plates to measure F1 specific antibodies in the serum by indirect ELISA.

A robust antibody response against the F1 antigen was successfully induced by EV76 immunization. Sera from all vaccinated mice (m1-m6), collected on day 30 post-immunization, showed high titers of F1-specific antibodies as measured by indirect ELISA (**Figure 1**). Positive signals were evident even at a 1:10^5^ serum dilution. Conversely, serum from the unvaccinated control mouse (m7) displayed no detectable binding to the F1 antigen at any dilution.

### 3.2 Building a murine scFv phage display library for the isolation of F1-specific antibodies

**Figure 2:**
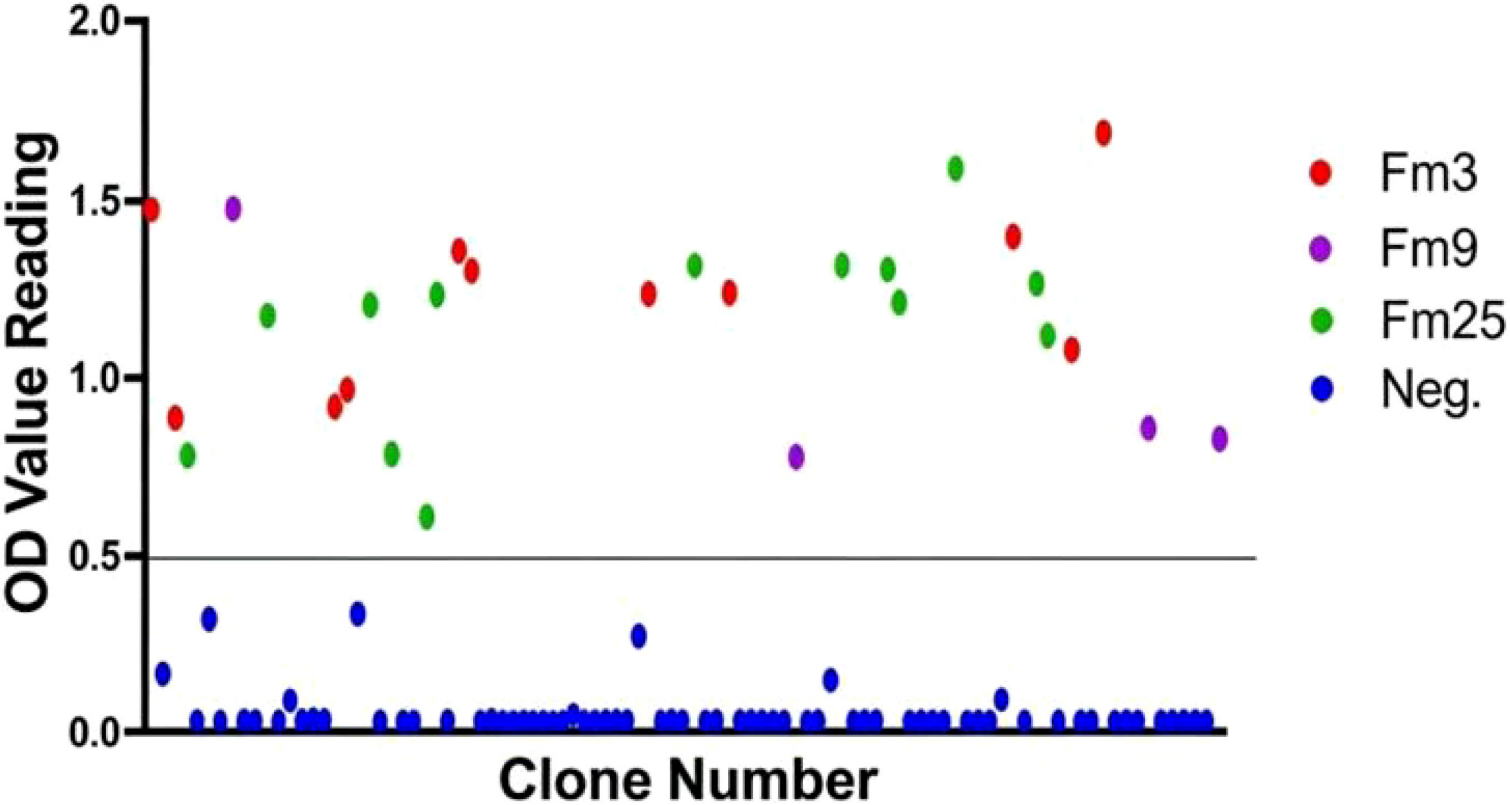
Panning ScFv antibodies from mice phage library. Phage ELISA was used to select ScFv antibodies against F1. F1 coated on 96-well plate at 200 ng per well, 96 samples of monoclonal phage supernatants were added to each well. HRP-conjugated anti-M13 mAbs were used as secondary antibody. After TMB color development, OD 450nm >0.5 were regarded as positive clones. Total 28 positive clones bind to F1 were sequenced, and variable gene sequences of heavy and light chains were run through the blast and aligned with homologous sequences of the IMGT (www.imgt.org/) database. Three unique mAbs, named Fm3, Fm9, and Fm25, were obtained. Neg. Negative.

Variable regions of the heavy and light chains (approximately 350 base pairs [bp]) were amplified from murine splenic cDNA. These fragments were then randomly assembled into single-chain variable fragments (scFv) using overlap extension PCR, generating products of approximately 750 bp. The purified scFv fragments were digested and cloned into a phage display vector. The ligated products were electroporated into *Escherichia coli* XL1-Blue competent cells to generate the primary antibody library. The library size was determined to be 2 × 10^8^ CFU. Sanger sequencing of 20 randomly selected clones confirmed that all 20 contained in-frame scFv inserts, demonstrating 100% cloning efficiency. Following three rounds of biopanning against the F1 antigen, 96 individual clones were randomly selected for analysis. After IPTG induction, phage-ELISA identified 28 positive clones exhibiting optical density at 450 nanometers (OD450) values exceeding 0.5 (**Figure 2**). The bacterial cultures of these 28 clones were sent for DNA sequencing. Sequence analysis and alignment via the IMGT database identified three unique mAbs, designated Fm3, Fm9, and Fm25. The Fm9 antibody was excluded from subsequent experiments due to failure in achieving soluble expression.

### 3.3 Specific *in vitro* binding of recombinant murine IgG antibodies to the F1 antigen

**Figure 3:**
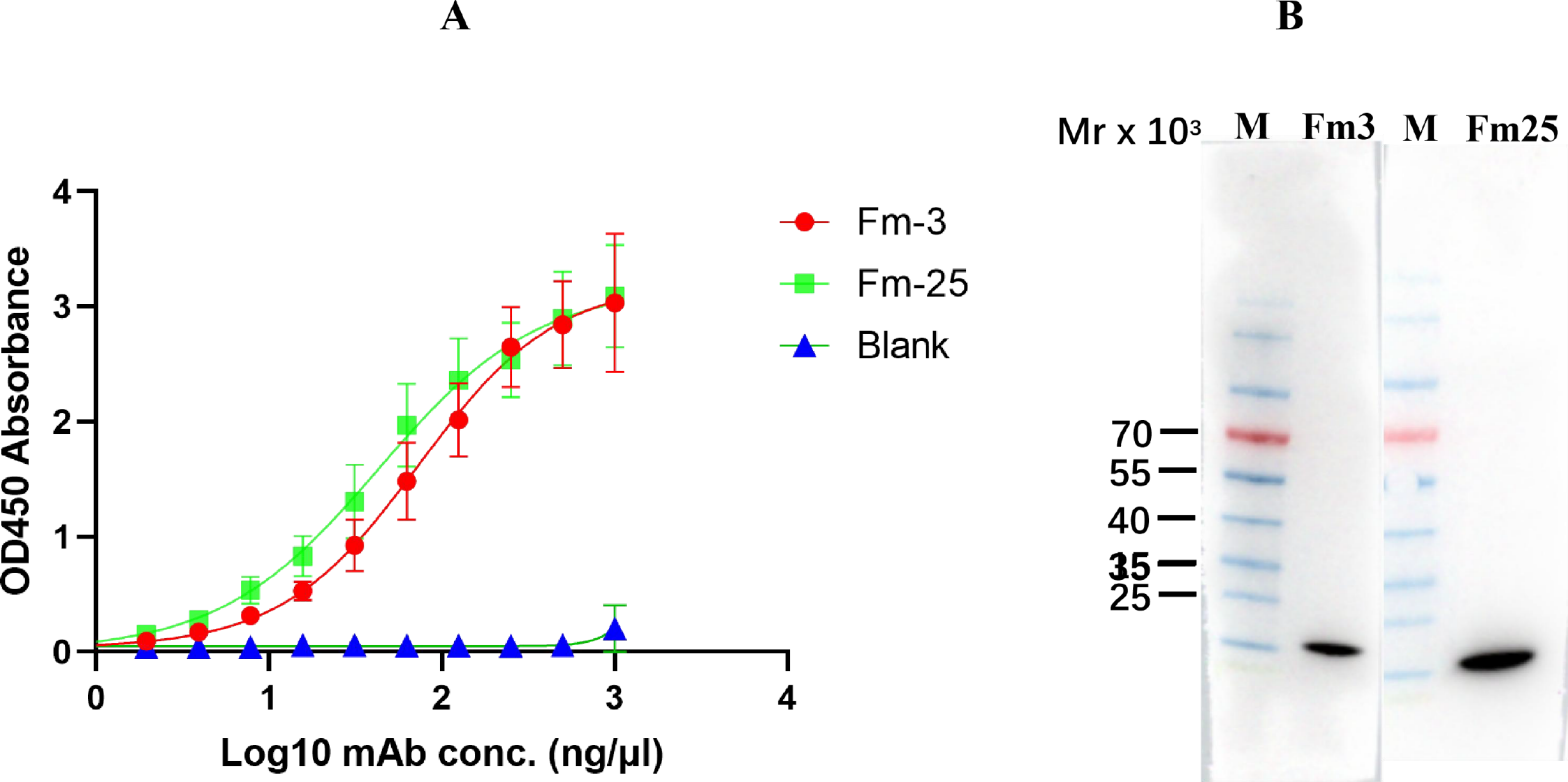
Characterization of the binding specificity of two monoclonal antibodies (mAbs) to the F1 antigen. **(A)** Indirect ELISA binding curves of 2 antibodies against F1. 96-well plate was coated with F1 protein. Purified mAbs were serial diluted as first antibody. HRP-conjugated goat anti-mouse was used as secondary antibody to deleopme the absorbance values at 450 nm. **(B)** Western blot analysis of antibodies using purified F1. F1 was electrophoresed and transferred to membranes and then probed by Fm3 and Fm25 antibodies, and secondary antibodies and visualized by ECL. M Denotes the pre-stained protein standard marker.

The binding specificity of Fm3 and Fm25 to the F1 antigen was evaluated using indirect ELISA and Western blot analysis (**Figure 3A**). Both Fm3 and Fm25, when bound to the F1 antigen, were effectively detected by an anti-mouse IgG secondary antibody, producing clear dose-response curves. Nonlinear regression analysis determined the half-maximal effective concentration (EC50) values to be 59 nanograms per microliter (ng/µL) for Fm3 and 34 ng/µL for Fm25. In the Western blot assay, both mAbs specifically recognized the denatured F1 protein, yielding a distinct band at approximately 15 kilodaltons (kDa) (**Figure 3B****).** These results confirms that Fm3 and Fm25 are conventional mAbs targeting linear epitopes.

### 3.4 Two mAbs exhibite high affinity and share a same epitope in F1 antigen

**Figure 4:**
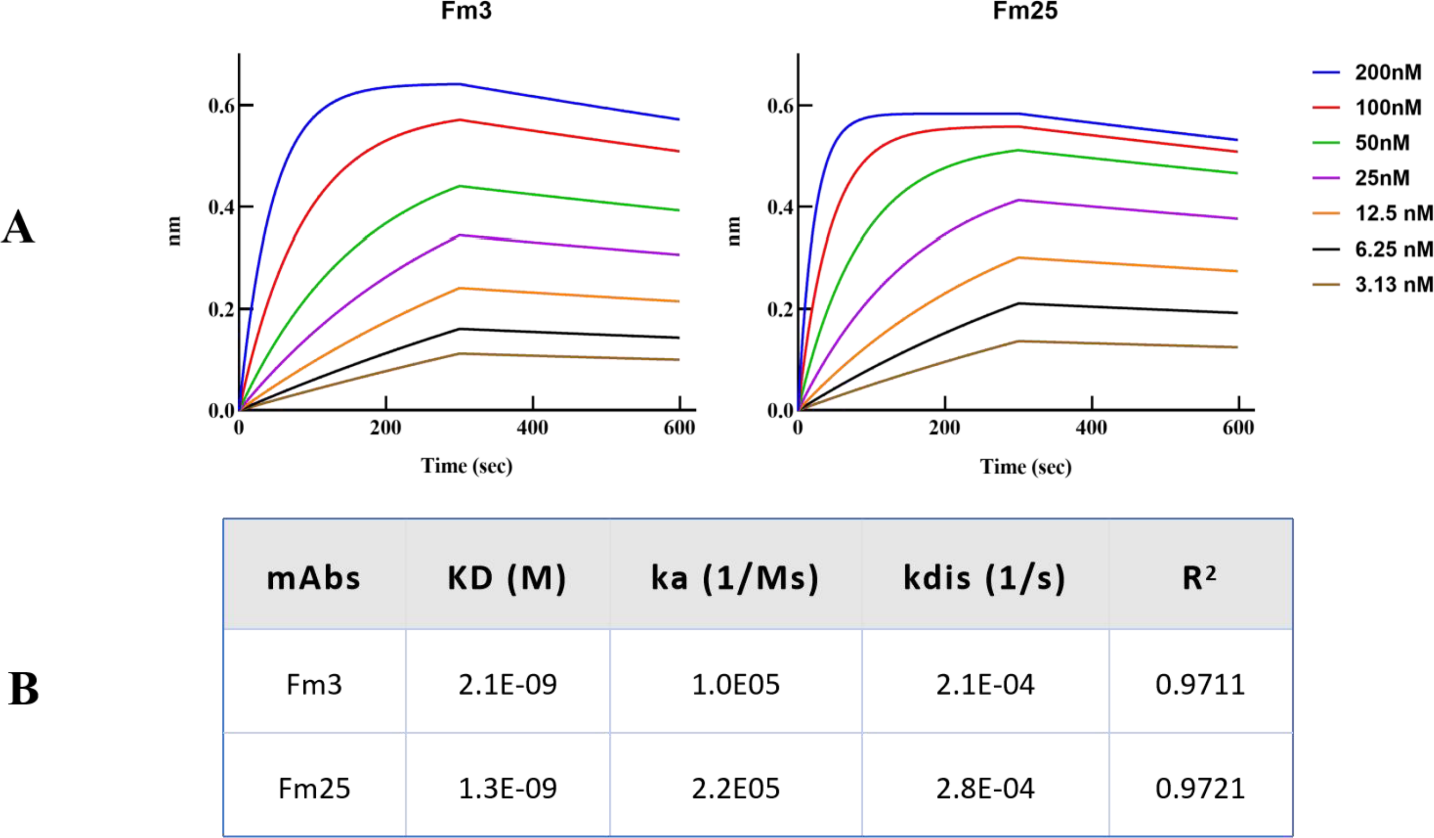
The kinetic analysis results of two mAbs against the F1 antigen. **(A)** Fm3 and Fm25 were immobilized onto ProA biosensors, association and dissociation curves against F1 of serial concentrations (from 200nm to 3.13 nm) were monitored in real-time by BLI. **(B)** Corresponding kinetic parameters for the interaction between the mAbs and F1. KD: Equilibrium dissociation constant, Ka: Association rate constant, kdis: Dissociation rate constant, R2: Goodness of fit.

The binding affinity of the two mAbs (Fm3 and Fm25) for the F1 antigen was quantified using bio-layer interferometry. Representative binding sensor-grams are shown. Both antibodies, at concentrations ranging from 200 nanomolar (nM) to 3.13 nM, produced clear concentration-dependent association and dissociation curves upon interaction with the F1 antigen (**Figure 4A****).** The kinetic parameters derived from this analysis are summarized in the corresponding table. The equilibrium dissociation constant (KD) was determined to be 2.1 nM for Fm3 and 1.3 nM for Fm25. These low nanomolar KD values confirm that both antibodies possess high affinity for their respective target (**Figure 4B****).**

**Figure 5:**
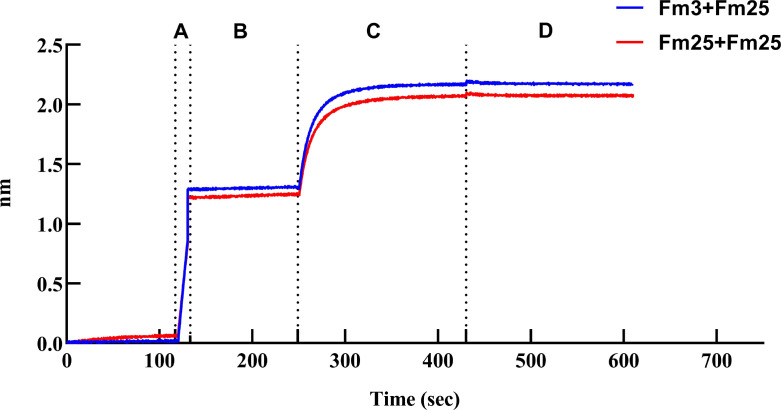
Binding competition of two monoclonal antibodies (mAbs) against the F1 antigen. Biotinylated F1 was loaded onto streptavidin (SA) sensors. Fm3 and Fm25 were associated to F1, then Fm25 was used for secondary to competed with Fm3 and Fm25. **(A)** antigen loading phase. **(B)** baseline phase. **(C)** first antibody association phase. **(D)** second antibody association phase.

The binding competition of Fm3 and F25 against F1 was performed by BLI. Biotinylated F1 was immobilized onto SA sensors (**Figure 5A**). Both Fm3 and Fm25 were able to associate to F1 on SA sensors in phase C. However, in phase D, Fm25 was used for secondary antibody. Fm25 did not trigger a more binding curve on immobilized Fm3. This suggests that Fm3 and Fm25 share the same epitope on F1.

### 3.5 Fm3 and Fm25 demonstrated potent efficacy, enabling survival following a lethal dose of the virulent *Y. pestis* 141 and S19960127

**Figure 6:**
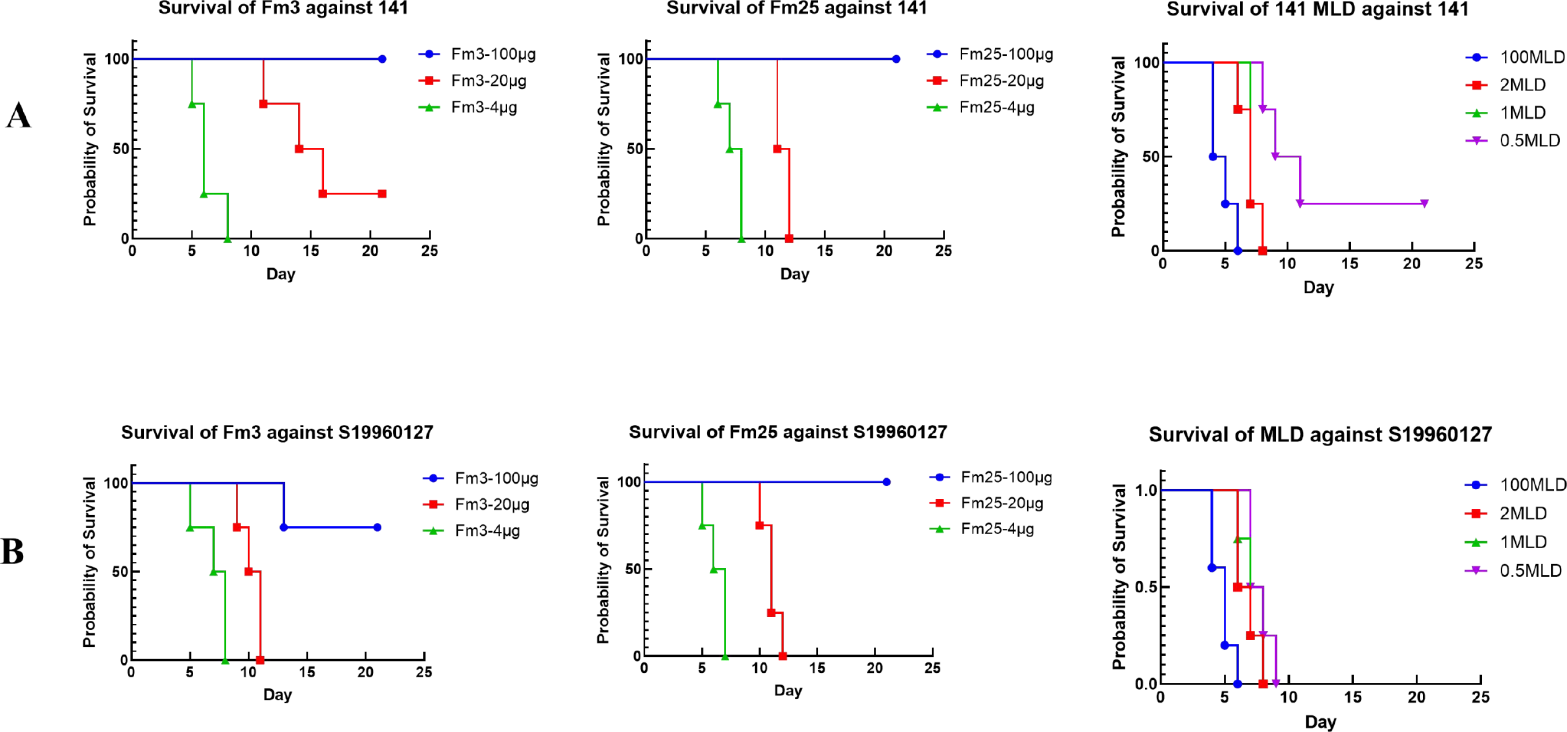
Survival curves of mice in *Y. pestis* challenge experiment. **(A)** Survival curves of mice following 100MLD challenge with *Y. pestis* 141. Mice were passively immunized with Fm3 or Fm25mAbs and challenged 24 hours later with 100 MLD of either the virulent *Y. pestis* 141. Survival was monitored for 21 days post-challenge. 100 µg group shows 100% protection. While, 20 µg,and 4 µg control groups indicate an absence of protective efficacy against *Y. pestis* 141. The right is the survival curves of mice challenged by a sequential dose of *Y. pestis* 141 (100, 2, 1 and 0.5 MLD) for bacterial back-titration **(B)** Survival curves of mice following lethal challenge with *Y. pestis* S19960127. A similar protective pattern was observed as challenged with *Y. pestis* 141.

Mice were passively immunized with Fm3 or Fm25 mAbs and challenged 24 hours later with 100 MLD of either the virulent *Y. pestis* 141 or S19960127. Survival was monitored for 21 days post-challenge. A dose of 100 µg of either Fm3 or Fm25 provided 100% protection against challenge with *Y. pestis* 141. In the 20 µg group, only Fm3 conferred partial protection, resulting in a 25% survival rate. All animals in the 4 µg groups succumbed within approximately 10 days, indicating an absence of protective efficacy at this low dosage. In the control groups challenged with varying MLD levels without antibody treatment, only one animal in the 0.5 MLD group survived through the end of the 21-day observation period. Organ imprint cultures from this survivor confirmed the clearance of *Y. pestis*, suggesting the establishment of a sublethal, self-resolving infection. All animals in the higher MLD control groups died within 10 days (**Figure 6A****).**

A similar protective pattern was observed in mice challenged with the *Y. pestis* S19960127. At a dose of 100 µg, Fm3 offered 75% protection, while the same dose of Fm25 provided complete (100%) protection with no mortality observed. In contrast, all animals in the 20 µg and 4 µg groups succumbed within approximately 10 days, indicating a lack of protective efficacy at these lower concentrations (**Figure 6B****).** In the control groups challenged with varying MLD of *Y. pestis* S19960127 (without antibody treatment), all animals died within 10 days, and no clear dose-response relationship in survival time was evident. This observation suggests that the actual virulence of the prepared *Y. pestis* S19960127 inoculum likely exceeded the theoretical 100 MLD, which accounts for the comparable survival times observed across the 0.5, 1, and 100 MLD challenge groups.

To confirm the cause of mortality, imprint cultures of heart, liver, spleen, and lung tissues from all deceased animals were performed on Hottinger agar plates. Bacterial colonies were observed on all plates the following day. Subsequent lysis by a *Y. pestis*-*specific* bacteriophage produced clear plaques, confirming that all mortalities were attributable to acute plague infection.

### 3.6 The statistical analyses of survival and time-to-death *in vivo*

During the 21-day observation period, following challenge with 100.0 MLD highly virulent plague bacilli, the survival rates of mice in the Fm3 groups were 100% (100.0 µg), 25% (20.0 µg), and 0% (4.0 µg), with average survival times of 21.00 days, 15.50 days, and 6.25 days, respectively. In the Fm25 groups at the same doses, the survival rates were 100% (100.0 µg), 0% (20.0 µg), and 0% (4.0 µg), with average survival times of 21.00 days, 11.50 days, and 7.25 days, respectively. When attacked with 100.0 MLD of drug-resistant strain, the survival rates in the Fm3 groups were 75% (100.0 µg), 0% (20.0 µg), and 0% (4.0 µg), with average survival times of 19.00 days, 10.25 days, and 7.00 days, respectively. Under challenge with the same drug-resistant strain, the survival rates in the Fm25 groups were 100% (100.0 µg), 0% (20.0 µg), and 0% (4.0 µg), with average survival times of 21.00 days, 11.00 days, and 6.25 days, respectively. Correlating the mAbs dose with mouse survival time by Spearman’s analysis revealed that, after infection with the 100 MLD virulent strain and drug-resistant strain, both Fm3 and Fm25 immunization doses showed a positive correlation with the average survival time of mice (P < 0.05).

## 4 Discussion

The management of plague has historically relied on streptomycin monotherapy. However, due to its considerable side effects, combination antibiotic regimens have become the current standard of care (41, 42). Despite this advancement, notable limitations persist, including nephrotoxicity and allergic reactions. It has been reported in the literature that *Y. pestis* strains show decreased susceptibility to multiple front-line antibiotics, including streptomycin, doxycycline, and ciprofloxacin (43, 44). With the continuous evolution of drug-resistant strains, traditional diagnostic and therapeutic approaches have become inadequate, and time-consuming drug susceptibility testing fails to meet the demands for early detection, diagnosis, and treatment of plague (45, 46). Regarding prophylaxis, no approved injectable plague vaccine is currently available in China, and a recombinant subunit vaccine from the Lanzhou Institute of Biological Products still in clinical trials (47, 48). Consequently, there is an urgent clinical need to develop safer and more effective therapeutic countermeasures against plague.

Although therapeutic mAbs have demonstrated remarkable efficacy across a broad spectrum of diseases (49–52), establishing themselves as a cornerstone of contemporary biomedical research, the therapeutic landscape for plague remains substantially different (53, 54). Neutralizing antibodies, which are critical indicators of vaccine efficacy (55), are severely understudied in the context of plague. To the best of our knowledge, only a limited number of such antibodies have been described in the literature, and the overall humoral immune response against *Y. pestis* remains poorly characterized. Among murine-derived mAbs, the first reported was F1-04-A-G1, an anti-F1 antibody described by the U.S. Army Medical Research Institute of Infectious Diseases in 1997 (56). This antibody conferred 100% protection in mouse models; however, its sequence information has not been disclosed to date. A second anti-F1 murine antibody, F2H5, was reported by Chen Wei’s group in 2017, with its humanized version providing complete protection in mice at a dose of 100 µg (57). In 2018, Wu Zhiyuan et al. reported another anti-F1 antibody, 4C6, which achieved 100% protection at 400 µg against a challenge of 100 MLD of the virulent *Y. pestis* strain (58). The MAb7.3 antibody, reported by United Kingdom researcher Jim Hill and colleagues, was the first protective antibody targeting the V antigen, binding to an epitope within amino acids 135-275 (59). Subsequent studies demonstrated a synergistic protective effect when MAb7.3 was used in combination with F1-04-A-G1 (60). Regarding human-derived antibodies, in 2010, Xiao et al. isolated one anti-F1 antibody (m252) and two anti-LcrV antibodies (m253, m254) from a naive human antibody library. The germline gene genotypes for m252 were VH1-2/VK1-16, while those for m253 and m254 were VH1-18/VK1–9 and VH3-43/VK1-27, respectively. Although m252 alone demonstrated some protective efficacy (with m253 and m254 offering no protection), a combination of all three antibodies achieved greater than 80% protection, though this was still inferior to the benchmark murine antibody F1-04-A-G1 (61). In another study, Lillo et al. used recombinant F1 protein to screen a yeast-display antibody library, yielding two human antibodies (αF1Ig 2 and αF1Ig 8) and demonstrating their potential for both diagnosing and treating *Y. pestis* infection (62). Although the methods employed for the preparation and screening of the two mAbs were generally similar to those reported in other studies, the two mAbs (Fm3 and Fm25) showed significantly higher individual protective efficacy than most reported antibodies *in vivo*. Notably, these two mAbs exhibited remarkable preventive and therapeutic potential against drug-resistant *Y. pestis.*

The present study evaluated the protective efficacy of two murine-derived anti-F1 mAbs (Fm3 and Fm25) in a BALB/c mouse model of plague. Passive immunization with these antibodies, followed 24 hours later by lethal challenge (100 MLD) with either the virulent *Y. pestis* 141 or a streptomycin-resistant isolate, revealed a clear dose-dependent protection. Against the virulent *Y. pestis* 141, a 100 µg dose of either Fm3 or Fm25 provided complete (100%) protection. In the challenge with the streptomycin-resistant strain, 100 µg of Fm3 conferred 75% protection, and the same dose of Fm25 afforded 100% survival. These results demonstrate a strong positive correlation between the antibody dose and both the mean survival time and survival rate. However, the number of mAbs that have been identified remains low. This phenomenon may be due to two reasons. The first reason is laboratory animals. Mice have received programmed immunization with excess antigen. When animals were sacrificed, *Y. pestis* induce a strong humoral immune response against F1. During the process of antibody affinity maturation, the affinity of antibodies increase gradually, while the diversity of antibody decreased. This may be one for the low diversity we obtained. Another factor is the biological bias present in phage display technology, which is an inherent feature of phage display libraries. This bias is typically introduced during sample selection, PBMCs isolation, library construction, phage packaging and library panning. All these can lead to the decrease in diversity.

The protective efficacy of mAbs against *Y. pestis* is closely associated with their antigen binding affinity, epitope characteristics, and the neutralizing ability against key virulence components. Based on the IgG isotype and the established literature on mAbs against extracellular bacterial pathogens, these mAbs may protect against *Y. pestis* via complement deposition and/or enhanced opsonophagocytosis by macrophages (63). In this study, the two obtained antibodies, Fm3 and Fm25 both showed high binding affinity to F1 antigen, which lays a solid foundation for their efficient *in vivo* protective effect. Passive immunization targeting critical virulence factors, particularly LcrV and F1, has been consistently shown to provide rapid and effective protection (64), even against pneumonic plague, when delivered systemically or directly via the pulmonary route (65, 66), but mAbs combined with subunit vaccines may cause epitope masking (67). Beyond LcrV and F1, other targets include YopM, Pla, and LPS (68–70), and fibrinolysin (Fib) (71). However, currently few neutralizing antibodies targeting these antigens have been developed, and their protective efficacy when used alone is generally not comparable to antibodies targeting F1 and LcrV. Protective anti-LcrV antibodies neutralize the type III secretion system by blocking Yop translocation (72). For anti-F1 antibodies, the mechanism of protection is not fully understood at present. Despite these advances, therapeutic mAbs for plague remain limited. To date, few protective mAbs have been reported, and most are murine-derived. Collectively, these insights support developing sequence-defined, single-agent antibodies against drug-resistant *Y. pestis*, as exemplified by murine anti-F1 mAbs Fm3 and Fm25, which show potent efficacy against virulent and streptomycin-resistant strains in mouse models. Furthermore, the *Y. pestis* S19960127 retained F1 antigen expression, which did not affect the antibody target’s validity. However, resistant strains significantly challenge plague treatment, as they undermine traditional antibiotics. The slow development of new antibiotics heightens the need for alternative treatments. This study showed that Fm3 and Fm25 effectively protect against drug-resistant infections, providing a new emergency treatment strategy for plague outbreaks. Despite the F1 target being unaffected by drug resistance, the protective efficacy of these mAbs against drug-resistant strains is crucial for clinical and public health.

In summary, this systematic evaluation of Fm3 and Fm25 provides critical preclinical data supporting their further development as therapeutic candidates for plague, offering valuable insights for future antibody-based treatments, the optimization of therapeutic formulations, and potential application in larger animal models, including non-human primates, which is essential for both public health and biosecurity preparedness.

## 5 Conclusion

In conclusion, we successfully generated two novel anti-F1 mAbs, Fm3 and Fm25, which demonstrated significant protection against virulent and drug-resistant strains of *Y. pestis*. Both mAbs conferred significant protection in mice against a lethal challenge with the virulent *Y. pestis* 141, with Fm25 achieved 100% protection. Crucially, Fm25 also exhibited substantial efficacy against a drug-resistant strain. These findings validate anti-F1 mAbs as promising immunotherapeutics for both antibiotic-sensitive and drug-resistant plague. Furthermore, this findings provides a foundation for the development of monoclonal antibody immunotherapeutics against both antibiotic-sensitive and drug-resistant plague, highlighting a promising avenue to address the limitations of current antibiotic regimens.

## Data Availability

The original contributions presented in the study are included in the article/**Supplementary Material**. Further inquiries can be directed to the corresponding authors.

## Supplementary material

Supplementary Table 1 Primer sequences for mouse immunoglobulin variable region (VH and VK) amplification.
