# Supplementary material for: Protective outcomes of high-affinity monoclonal antibodies against drug-resistant plague strains
Source: Front Immunol. 2026 Jun 23;17:1835695. doi: 10.3389/fimmu.2026.1835695 (PMC13337711; doi:10.3389/fimmu.2026.1835695)
Supplement: Supplementary Table 1 — Primer sequences for mouse immunoglobulin variable region (VH and VK) amplification. [file Table1.doc]

**Supplementary Table S1 Primer sequences for mouse immunoglobulin variable region (VH and VK) amplification**

| **Primer Nomenclature** | **Sequence(3’⁓5’)** |
| --- | --- |
| MVH1 | GGTGGTTCCTCTAGATCTTCCCTCGAVGTGAAGCTSGTGGAGTCT |
| MVH2 | GGTGGTTCCTCTAGATCTTCCCTCGATGTGAACYTGGAAGTGTCT |
| MVH3 | GGTGGTTCCTCTAGATCTTCCCTCGATGTRCAGCTTCAGGAGTCA |
| MVH4 | GGTGGTTCCTCTAGATCTTCCCTCGAGWTCCAGCTGCARCAGTCT |
| MVH5 | GGTGGTTCCTCTAGATCTTCCCTCGAAGTGAARMTTGAGGAGTCA |
| MVH6 | GGTGGTTCCTCTAGATCTTCCCTCGAGGTYCAGCTBCARCAGTCT |
| MVH7 | GGTGGTTCCTCTAGATCTTCCCTCGAGGTGCAGCTKGTKGAGWCT |
| MVH8 | GGTGGTTCCTCTAGATCTTCCCTCGAYGTGAWGCTGGTGGARTCT |
| MVH9 | GGTGGTTCCTCTAGATCTTCCCTCGARGTSCAGCTGWWGSAGRCT |
| MVH10 | GGTGGTTCCTCTAGATCTTCCCTCGAGGTVCAGCTYCAGSAGTCR |
| MVH11 | GGTGGTTCCTCTAGATCTTCCCTCGARGTCCAGCTGCAACARTCT |
| MVH12 | GGTGGTTCCTCTAGATCTTCCCTCGARGTGCAGCTGGTGGARTCT |
| MVH13 | GGTGGTTCCTCTAGATCTTCCCTCCAGGCCCARMTRCAGCAGYCT |
| MVHS1 | GGTGGTTCCTCTAGATCTTCCCTCGAGGTCMDGCTGCARCAGTCT |
| MVHS2 | GGTGGTTCCTCTAGATCTTCCCTCGAGGTGARGCTGRTGGAATCT |
| MSCG1ab-B | CTTGGCCGGCCTGGCCACTAGTGACAGATGGGGSTGTYGTTTTGGC |
| MSCG3-B | CCTGGCCGGCCTGGCCACTAGTGACAGATGGGGCTGTTGTTGT |
| MSCM-B | CCTGGCCGGCCTGGCCACTAGTGACATTTGGGAAGGACTGACTCTC |
| MVK1 | GGGCCCAGGCGGCCGAGCTCGACATCMWGMTGACCCARTCT |
| MVK2 | GGGCCCAGGCGGCCGAGCTCGACATYCAGATGACHCARTCT |
| MVK3 | GGGCCCAGGCGGCCGAGCTCGACATYTTGRTGACYCAGTCT |
| MVK4 | GGGCCCAGGCGGCCGAGCTCGACATTGTGCTVACMCARTCT |
| MVK5 | GGGCCCAGGCGGCCGAGCTCGACATTGTGATGWCHCAGTCT |
| MVK6 | GGGCCCAGGCGGCCGAGCTCGATATTGTGATGACKCAGGCT |
| MVK7 | GGGCCCAGGCGGCCGAGCTCGAYGTTGTGATGACCCARACT |
| MVK8 | GGGCCCAGGCGGCCGAGCTCGAAATTGTGYTSACYCAGTCT |
| MVK9 | GGGCCCAGGCGGCCGAGCTCGAAAWKGTKCTCACCCAGTCT |
| MVK10 | GGGCCCAGGCGGCCGAGCTCCAAATTSTTCTCWCCCAGTCT |
| MVK11 | GGGCCCAGGCGGCCGAGCTCAGYATTGTGATGACCCAGWCT |
| MVK12 | GGGCCCAGGCGGCCGAGCTCGATATCCAGATGACACAGACT |
| MSCJK12-B | GGAAGATCTAGAGGAACCACCTTTKATTTCCAGYTTGGTCCC |
| MSCJK4-B | GGAAGATCTAGAGGAACCACCTTTTATTTCCAACTTTGTCCC |
| MSCJK5-B | GGAAGATCTAGAGGAACCACCTTTCAGCTCCAGCTTGGTCCC |
